# Supplementary material for: Circulating mucosal-associated invariant T cells identify patients responding to anti-PD-1 therapy
Source: Nat Commun. 2021 Mar 15;12:1669. doi: 10.1038/s41467-021-21928-4 (PMC7961017; doi:10.1038/s41467-021-21928-4)
Supplement: Supplementary file 1 — Supplementary Information [file 41467_2021_21928_MOESM1_ESM.pdf]

Supplementary information

De Biasi et. al.

Circulating mucosal associated invariant T cells identify patients responding to anti-PD1 therapy

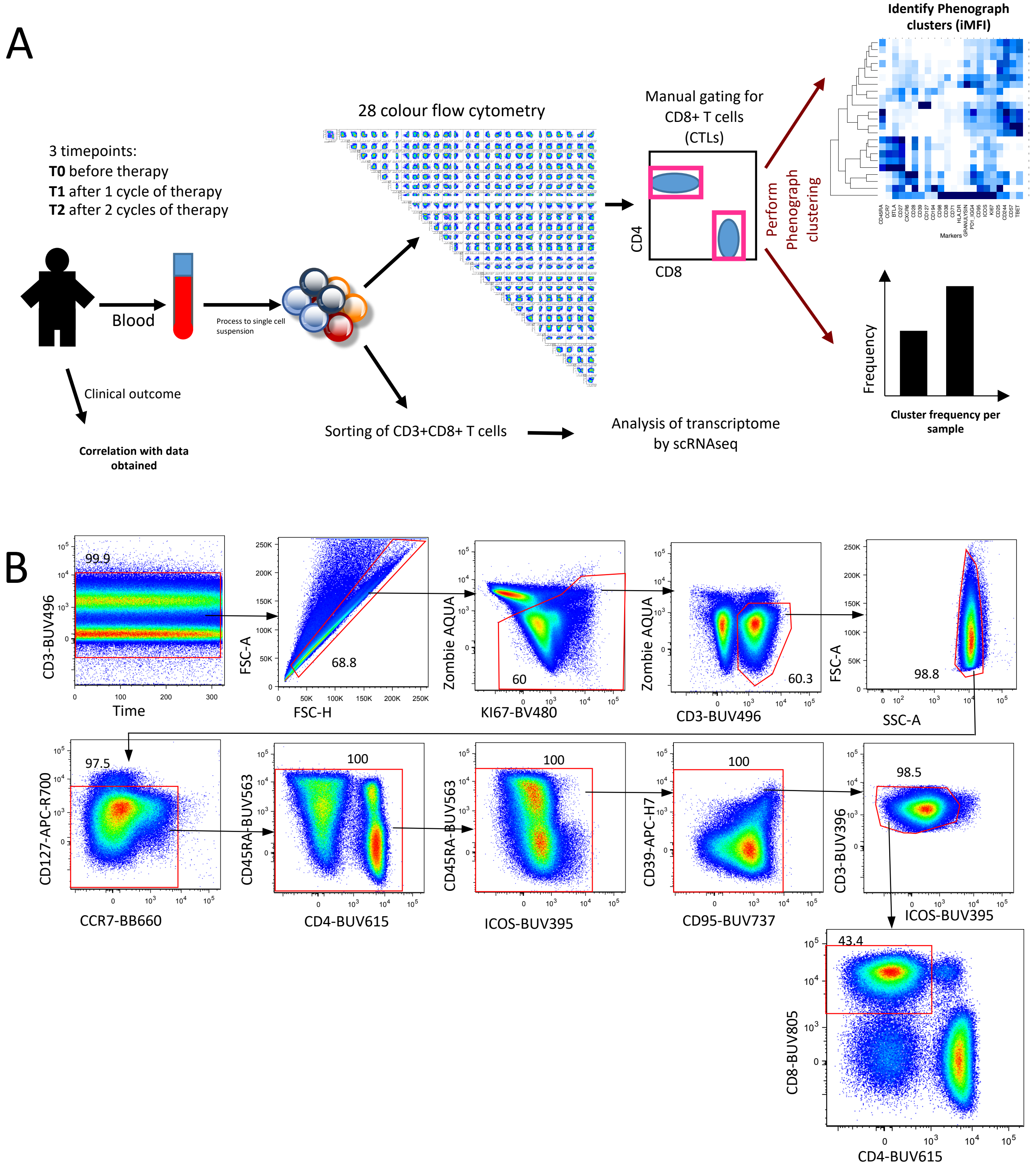

Supplementary Figure 1. Experimental workflow and gating strategy for the identification of CD8⁺ T cells. A. Experimental workflow on PBMC isolated from patients with metastatic melanoma. Blood was obtained before therapy (T0) and after 1 and 2 cycles of therapy (T1 and T2, respectively). Then, PBMC were isolated to perform 28-colour flow cytometry and downstream analysis. CD3⁺CD8⁺ T cells were sorted for single-cell RNA sequencing (scRNA-seq) B. Gating strategy used to identify CD8+ T cells. A first gate was set on the parameter «time». Then, doublets and dead cells were removed. CD3⁺ cells were selected and all unwanted cells were removed from the analysis (not lymphocytes according to physical parameters, all flouorochrome aggregates (middle row). Finally, in this cleaned subset of T cells, CD8⁺ T cells were recognized and used for unsupervised analysis.

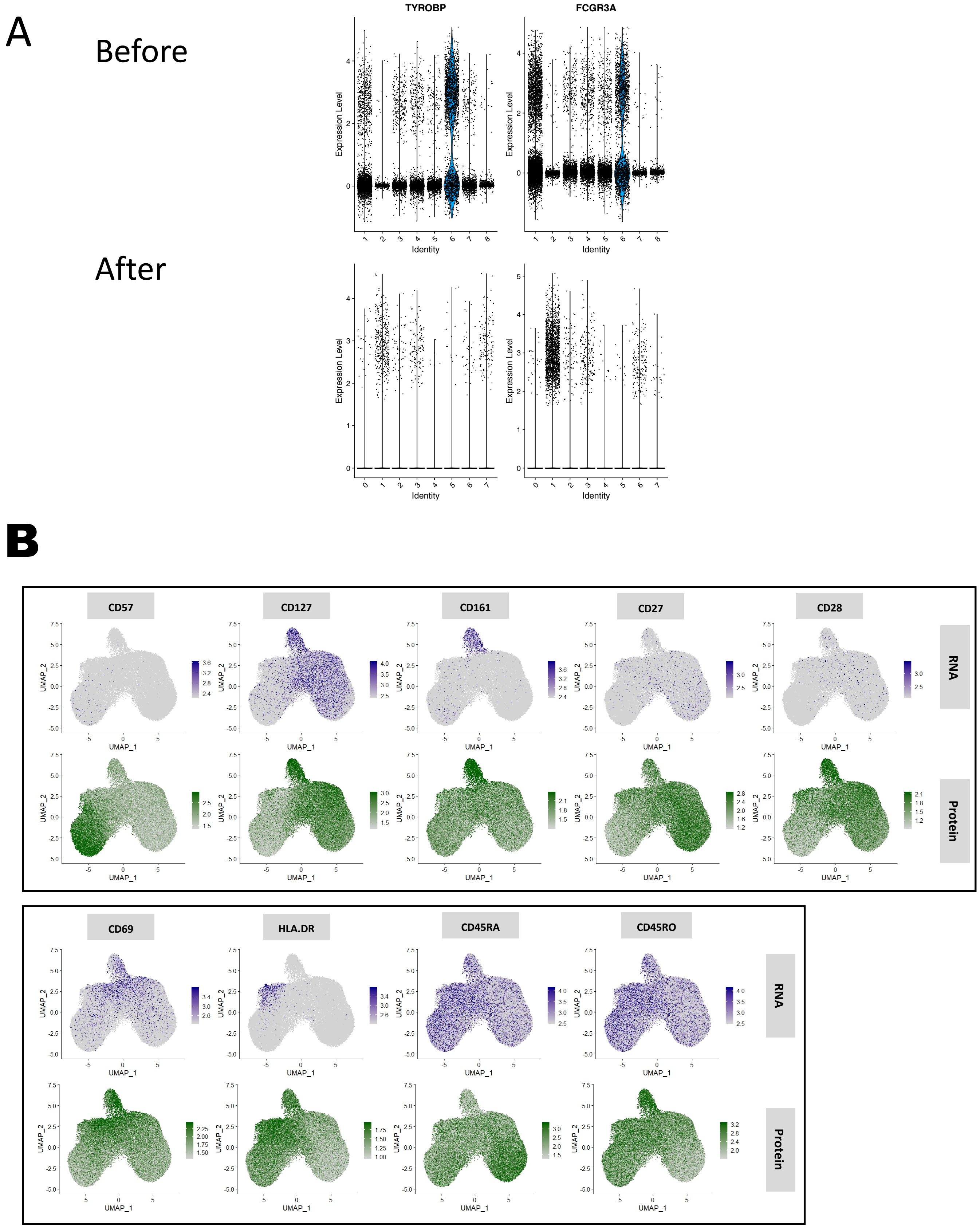

Supplementary Figure 2. Gene expression analysis for scRNA-seq.
A. Gene expression analysis of *TYROBP and FCGR3A* before and after the removal of NK cells. B. Neural network analysis run after the removal of NK cells. This analysis compares gene and protein expression. The most representative genes expressed by CD8+ T cells are shown.

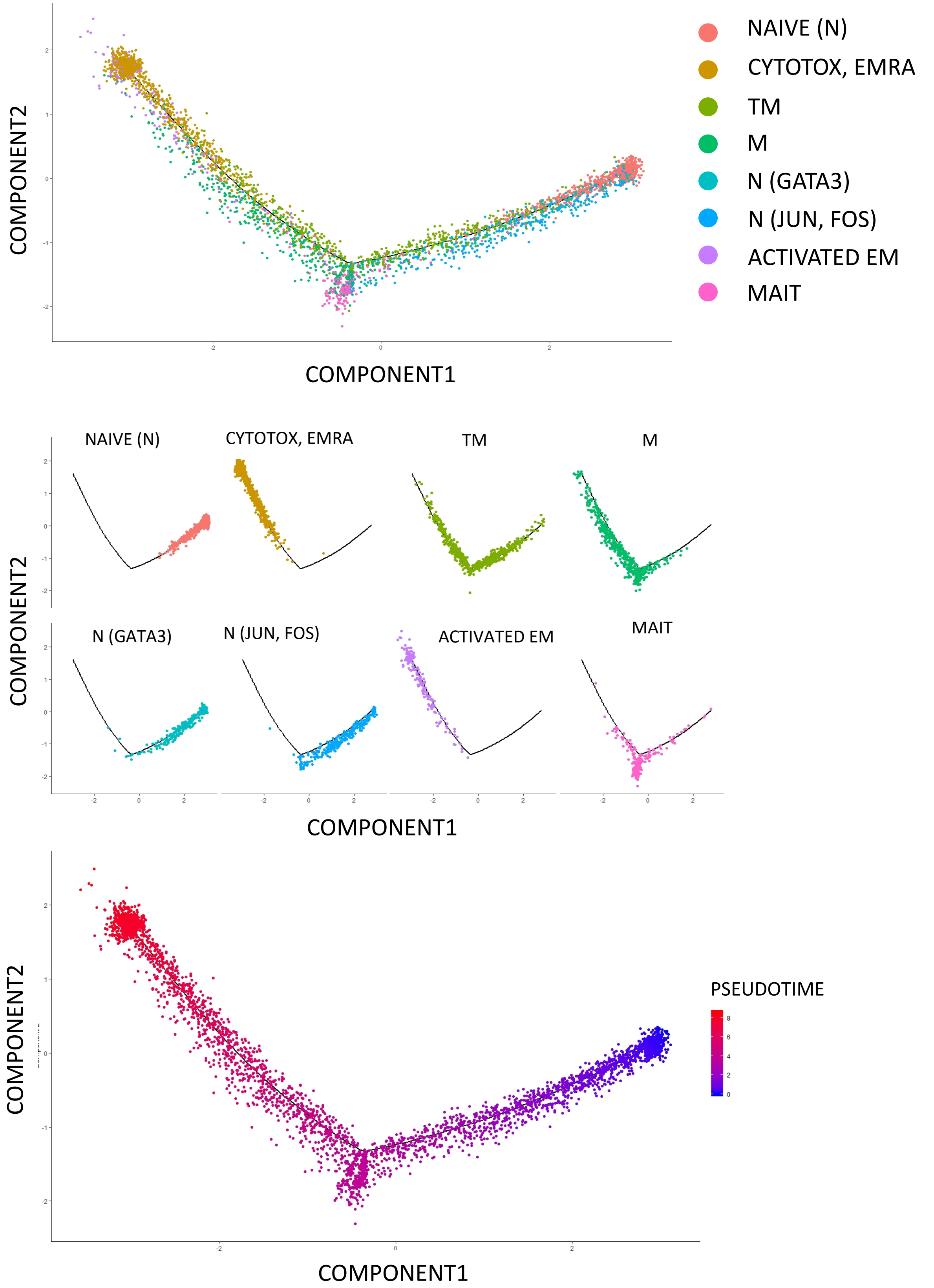

Supplementary Figure 3. Trajectory analysis for CD3⁺CD8⁺ T cell clusters.

Trajectory analysis for the eight CD3⁺CD8⁺ T cells clusters. Cell expression profiles in a two-dimensional independent space. Solid black line indicates the main diameter path of the minimum spanning tree (MST) and provides the backbone of Monocle's pseudotime ordering of the cells. Each dot represents an individual cell colored by cluster or by pseudotime.

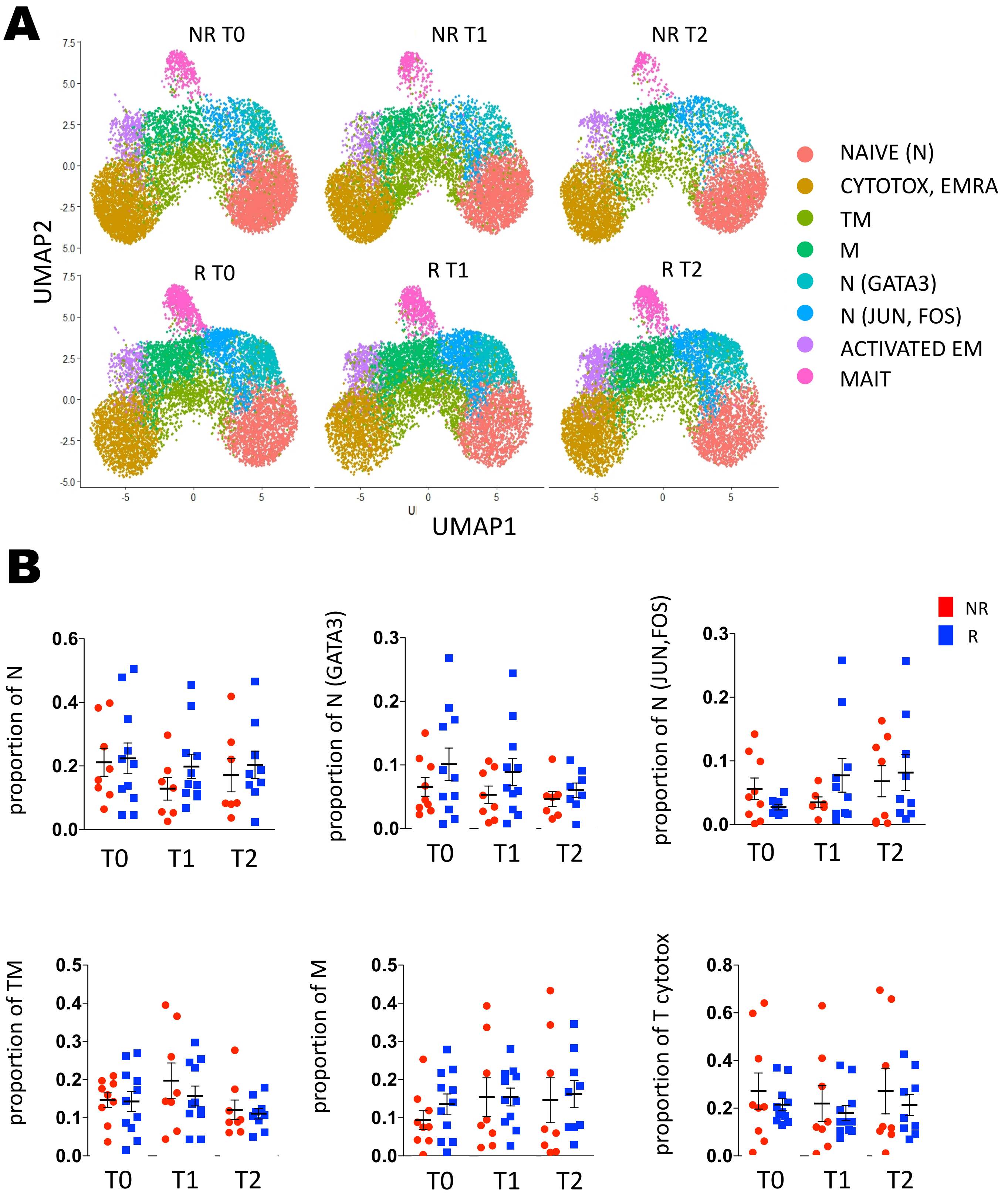

Supplementary Figure 4. Clusters' distribution during therapy.

A. Distribution of different cell clusters identified by sc-RNAseq analysis. B. Proportion of different cell clusters identified by sc-RNAseq analysis. Data represent individual values, mean (centre bar) ± SEM (upper and lower bars).

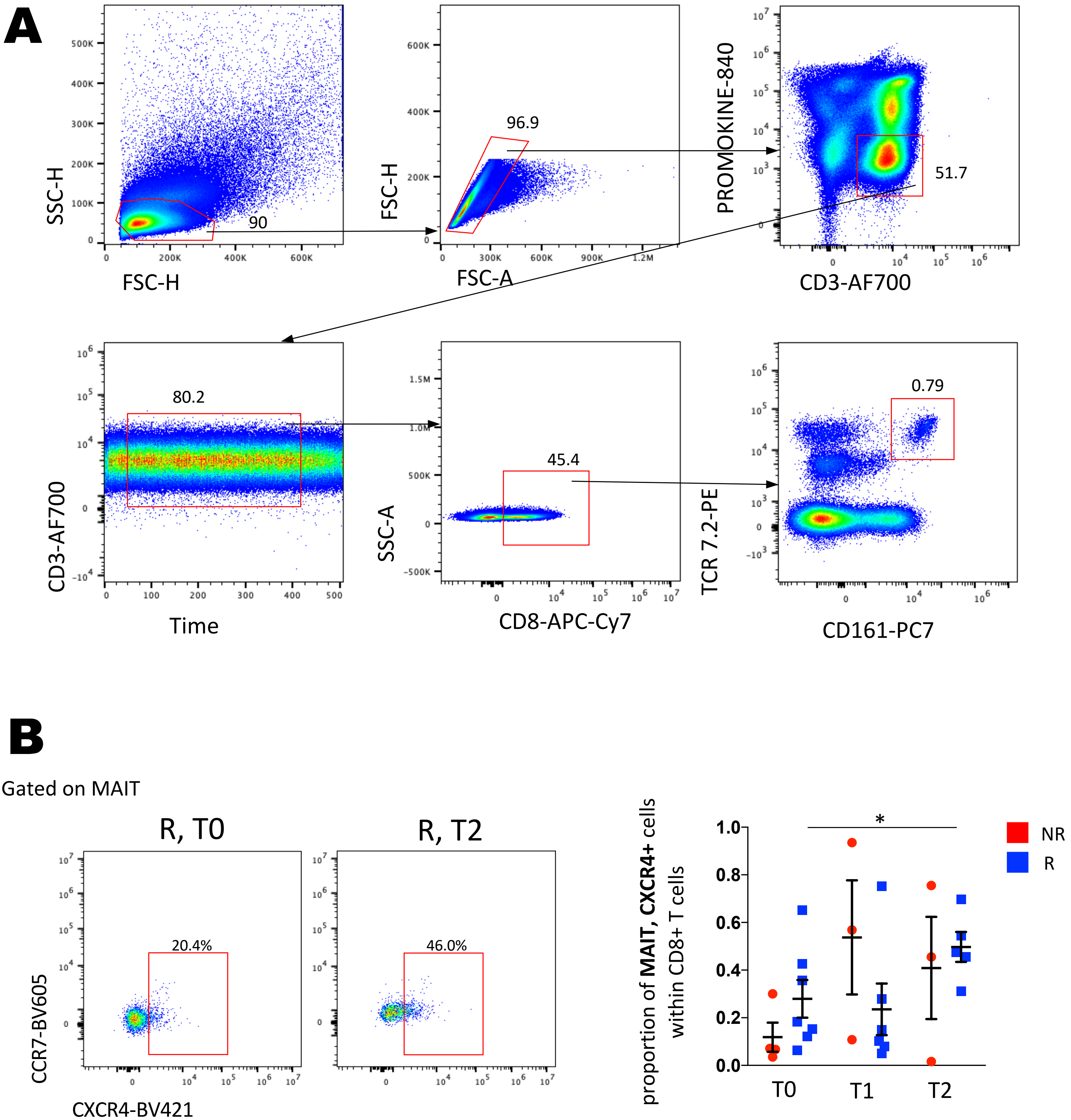

Supplementary Figure 5. Gating strategy for MAIT identification.

A. Gating strategy used for the analysis of MAIT cells. Cells were first selected on the basis of physical parameters, then doublets and dead cells were removed. $CD3^+$ cells were selected. Among them, $CD8^+$ T cells were identified. Within $CD8^+$ T cells, the expression of high levels of CD161 and TCR 7.2 identified MAIT cells. B. Representative dot plots of MAIT cells expressing CXCR4 of one R analysed at T0 (left) and T2 (central panel). On the right, proportion of MAIT cells expressing CXCR4 between R and NR at T0, T1, T2. Data represent individual values, mean (centre bar) ± SEM (upper and lower bars). Statistical test by Mann-Whitney nonparametric test, Bonferroni's multiple comparisons test; *p=0.045

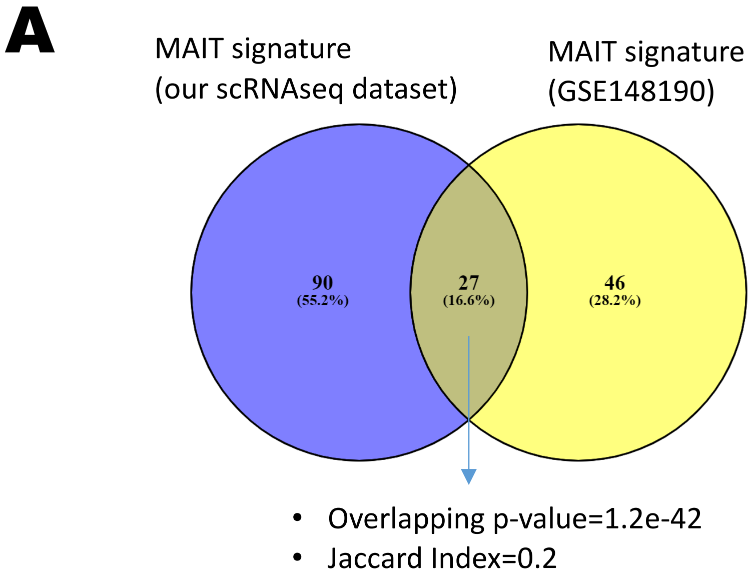

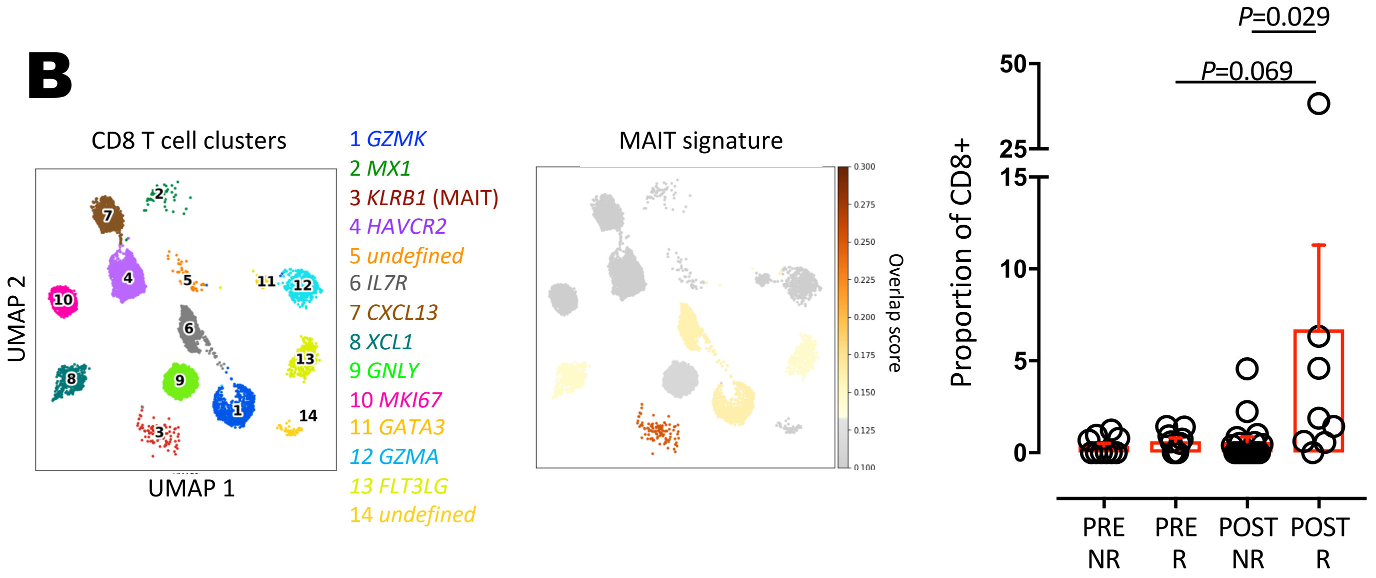

Supplementary Figure 6. MAIT signature in tumour infiltrating lymphocytes (TIL) dataset.
A. MAIT signature in TIL dataset. MAIT signature was confirmed by using GeneOverlap. In blue circle (left) numbers (percentage) of genes identifying MAIT cluster in our scRNAseq dataset; in yellow circle (right) numbers (percentage) of genes identifying MAIT cluster in GSE148190. The intersection between the two circles represent the number of genes (percentage) shared by the MAIT signature found in the two different datasets. B. Left: UMAP representation of CD8 T cell clusters from scRNA-seq data of melanoma R and NR to ICB (GSE120575). Center: overlap score of MAIT signature from Fig. 1B. Cluster 3 is the one with the highest score, thus identifying MAIT cells. Right: Relative proportion (% among CD8) of cluster 3 of MAIT cells in R and NR pre and post ICB. P values were calculated using non-parametric Kruskal-Wallis test and Dunn's multiple comparisons.

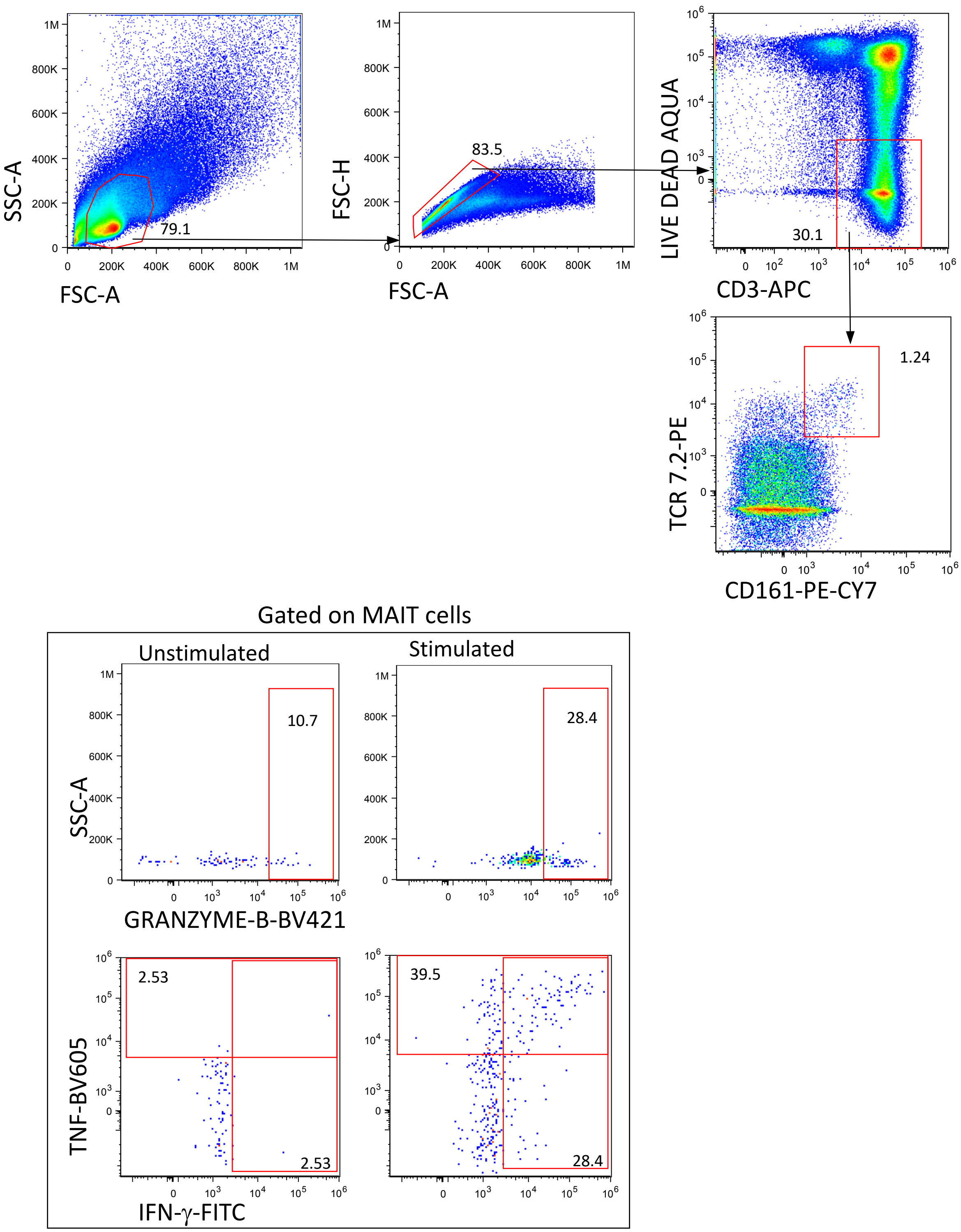

Supplementary Figure 7. Gating strategy for analysing cytokine production by MAIT cells.
Gating strategy used for the analysis cytokine production by MAIT cells. Cells were first selected on the basis of physical parameters, then doublets and dead cells were removed. $CD3^+$ cells were selected. Among them, MAIT cells were identified on the basis of the expression of CD161 and TCR 7.2. The production of granzyme B, tumour necrosis factor (TNF) and interferon-γ (IFN-γ) was quantified.

Specificity	Dye	Clone	Vendor	Catalog Number	Lot number	µl used (in a volume of 100µl)
AQUA	BV510	N/A	Biolegend	423102	B244918	0.06
CCR7	BB660	150503	BD	625454	7136810	1.25
CXCR6	BV421	13B 1E5	BD	566008	7131852	2.50
CD194	PE-CF594	1G1	BD	565391	7276847	0.30
CD25	BB790	M-A251	BD	353736	7150757	2.5
CD244	PC5.5	C1.7	BC	B21171	16	1.25
CD98	BB515	UM7F8	BD	565103	7283973	1.25
CD28	BV786	CD28.2	Biolegend	302950	B249581	0.6
CD39	APC-H7	A1	Biolegend	328226	B246491	1.25
CD127	APC-R700	HIL-7R-M21	BD	565185	7221803	2.5
CD38	BV711	HIT2	Biolegend	303528	B238633	2.5
CD272 (BTLA)	BV650	J168-540	BD	564803	7103949	0.60
CD57	BV605	NK-1	BD	563895	7018755	1.25
CD27	BV570	O323	Biolegend	302825	B244350	1.25
CD71	PE-CY5	M-A712	BD	551143	7058789	0.60
CD8	BUV805	SK1	BD	564912	7311599	1.25
CD95	BUV737	DX2	BD	564710	7032582	0.63
HLA-DR	BUV661	G46-6	BD	565073	7249926	0.03
CD4	BUV615	SK3	BD	624297		0.31
CD45RA	BUV563	HI100	BD	565702	7219651	1.25
CD3	BUV496	UCHT1	BD	564809	7312719	2.50
ICOS CD278	BUV395	DX29	BD	564777	7263764	2.50
PD-1	PE	EH12.1	BD			2.5
IgG4	PE	HP6025	Southern Biotech	9200-09	B3317-YD17B	1.25
TBET	PE-CY7	4B10	eBioscience	25-5825-80	4286798	0.15
GRANULYSIN	APC	DH2	Biolegend	348006	B192033	5.00
KI67	BV480	B56	BD	566109	7254647	5.00

Supplementary Table 1: Reagents for the 28-color panel used for the characterization of T cells.

Specificity	Dye	Clone	Vendor	Catalog Number	Lot Number	µl used in a volume of 100µl
**PromoFluor-840**	Maleimide	N/A	Promokine	PK-PF840-3-05	429P017	0.3
**CXCR4**	BV421	12G5	BD	562448	4003566	1.25
**CCR7**	BV605	G043H7	Biolegend	353224	B284011	2.5
**CD3**	AF700	UCHT1	Biolegend	300424	B279942	0.6
**CD8a**	APC-CY7	RPA-T8	Biolegend	301016	B274260	1.5
**CD161**	PC7	B30631	Beckman Coulter	B191B8	200023	2.5
**TCR72**	PE	3C10	Biolegend	351706	B292236	1.25
**CD69**	FITC	FN50	Biolegend	310904	B290844	0.6
**CD95**	BUV395	DX2	BD	740306	9269965	0.6
**CD38**	BUV496	HIT2	BD	564658	8339559	1.25
**HLA-DR**	BUV661	G46-6	BD	565073	7249926	0.3
**CD127**	BV650	A019D5	Biolegend	351325	B238890	0.6
**CD25**	BV785	BC96	Biolegend	302638	B237289	1.25
**CD45RO**	PC5.5	UCHL1	Beckman Coulter	B30638	200022	10
**Ki67**	BV480	B56	BD	566109	7254647	5
**GRANULYSIN**	AF647	DH2	Biolegend	348006	B192033	5

Supplementary Table 2. mAbs used in flow cytometry panel used for the detection of MAIT cells.

Specificity	Dye	Clone	Vendor	Catalog Number	Lot Number	μl used in a volume of 100μl
**Live Dead**	AQUA	N/A	ThermoFisher	L34965	2069643	1.25
**CD4**	AF700	RPA-T4	Biolegend	300526	B274110	0.6
**CD8**	APC-Cy7	RPA-T8	Biolegend	301016	B274260	0.6
**CD161**	PE-Cy7	HP-3G10	Biolegend	339918	B204086	0.6
**TCR 7.2**	PE	3C10	Biolegend	351706	B292236	1.25
**CD3**	PE-Cy5	UCHT1	Biolegend	300410	B252119	0.6
**TNF**	BV605	MAb11	Biolegend	502936	B282176	3.75
**IFN-γ**	FITC	B27	Biolegend	506504	B286029	2.5
**GRZB**	BV421	QA18A28	Biolegend	396414	B296875	2.5
**GRZA**	APC	CB9	Biolegend	507220	B277773	5

Supplementary Table 3. mAbs used for investigating the function of MAIT cells.
