# Supplementary material for: Circulating mucosal-associated invariant T cells identify patients responding to anti-PD-1 therapy
Source: Nat Commun. 2021 Mar 15;12:1669. doi: 10.1038/s41467-021-21928-4 (PMC7961017; doi:10.1038/s41467-021-21928-4)
Supplement: Supplementary file 3 — Reporting Summary [file 41467_2021_21928_MOESM3_ESM.pdf]

nature research

Corresponding author(s): Andrea Cossarizza, Sara De Biasi

Last updated by author(s): Feb 5, 2021

Reporting Summary

Nature Research wishes to improve the reproducibility of the work that we publish. This form provides structure for consistency and transparency in reporting. For further information on Nature Research policies, see our Editorial Policies and the Editorial Policy Checklist.

Statistics

For all statistical analyses, confirm that the following items are present in the figure legend, table legend, main text, or Methods section.

n/a	Confirmed	
☐	☒	The exact sample size (*n*) for each experimental group/condition, given as a discrete number and unit of measurement
☐	☒	A statement on whether measurements were taken from distinct samples or whether the same sample was measured repeatedly
☐	☒	The statistical test(s) used AND whether they are one- or two-sided<br>*Only common tests should be described solely by name; describe more complex techniques in the Methods section.*
☒	☐	A description of all covariates tested
☐	☒	A description of any assumptions or corrections, such as tests of normality and adjustment for multiple comparisons
☐	☒	A full description of the statistical parameters including central tendency (e.g. means) or other basic estimates (e.g. regression coefficient) AND variation (e.g. standard deviation) or associated estimates of uncertainty (e.g. confidence intervals)
☐	☒	For null hypothesis testing, the test statistic (e.g. *F*, *t*, *r*) with confidence intervals, effect sizes, degrees of freedom and *P* value noted<br>*Give P values as exact values whenever suitable.*
☒	☐	For Bayesian analysis, information on the choice of priors and Markov chain Monte Carlo settings
☐	☒	For hierarchical and complex designs, identification of the appropriate level for tests and full reporting of outcomes
☒	☐	Estimates of effect sizes (e.g. Cohen's *d*, Pearson's *r*), indicating how they were calculated

Our web collection on statistics for biologists contains articles on many of the points above.

Software and code

Policy information about availability of computer code

Data collection	Analysis of T cells. A 30 parameter/28-color flow cytometry panel was optimized to broadly characterizes T cell differentiation and activation. Cryopreserved samples were thawed in R10 medium, i.e., RPMI supplemented with 10% foetal bovine serum (FBS), 100 U/mL penicillin, 100 µg/mL streptavidin, 2 mM L-glutamine, 20 mM HEPES (ThermoFisher, Eugene, OR) and 20 µg/ml DNase I from bovine pancreas (Sigma-Aldrich, St. Louis, MO). After washing with phosphate buffer saline (PBS), cells were stained immediately with the Zombie Aqua Fixable Viability kit (BioLegend, San Diego, CA) for 15 min at room temperature. Then, cells were washed and stained with the combination of monoclonal antibodies (mAbs). Intracellular detection of Ki-67, granulysin and T-bet was performed following fixation of cells with the FoxP3 transcription factor staining buffer set (eBioscience/ThermoFisher) according to manufacturer's instructions and by incubating with specific mAbs for 30 min at 4°C. Samples were acquired on a FACS Symphony A5 flow cytometer (BD Biosciences) equipped with five lasers (UV, 350 nm; violet, 405 nm; blue, 488; yellow/green, 561 nm; red, 640 nm) and capable to detect 30 parameters. Flow cytometry data were compensated in FlowJo by using single stained controls (BD Compbeads incubated with fluorochrome-conjugated antibodies).<br>A 18 parameter/16-color flow cytometry panel was then optimized to broadly investigate specifically mucosal invariant associated T (MAIT) cell phenotype. Cryopreserved samples were thawed and stained immediately with PromoFluor-840, viability probe (PromoCell - PromoKine) for 20 min at room temperature. Then, cells were washed and stained with the combination of mAbs purchased from either BD Biosciences, BioLegend, or eBioscience. Intracellular detection of Ki-67 and granulysin was performed following fixation of cells with the FoxP3/ transcription factor staining buffer set (eBioscience, ThermoFisher) according to manufacturer's instructions and by incubating with specific mAbs for 30 min at 4°C. Samples were acquired on a Cytoflex LX flow cytometer (Beckman Coulter, Hialeah, FL) equipped with six lasers (UV, 355 nm; violet, 405 nm; blue, 488; yellow/green, 561 nm; red, 638 nm; IR, 808nm) and capable to detect 21 parameters. Flow cytometry data were compensated in FlowJo by using single stained controls.<br>PBMC were rested for 4 hours at 37°C and then in vitro stimulated with anti-CD3/CD28 (Miltenyi, Bergisch Gladbach, Germany) and suboptimal concentration of IL-12 (Miltenyi) and IL-18 (R&D System, Minneapolis, MN) and a combination of those. A 11 parameter/10-color flow cytometer panel was optimized to identify MAIT cells producing Granzyme (GRZM)-A, GRZM-B, TNF and IFN-g that were detected after 16 hours of incubation. For the quantification of intracellular cytokines, cells were fixed with BD Cytofix/Cytoperm Fixation/Permeabilization Solution kit (BD Biosciences) according to the manufacturer's instructions. Samples were acquired on an Attune NxT acoustic flow cytometer

(ThermoFisher) equipped with four lasers (violet, 405 nm; blue, 488; yellow/green, 561 nm; red, 640 nm) and capable to detect 14 parameters. Flow cytometry data were compensated in FlowJo by using single stained controls as above.
For single cell RNA sequencing, samples were thawed in R10 supplemented with 20 µg/ml DNase I from bovine pancreas (Sigma-Aldrich). After washing with phosphate buffer saline (PBS), cells were stained with the Red Live Dead Fixable Viability kit (ThermoFisher) for 15 min at room temperature. PBMC were washed with PBS and stained with mAb anti-CD3-PE and -CD8-FITC. Viable CD3+,CD8+ T cells were sorted by using eS3 sorter (Bio-Rad Laboratories, Hercules, CA) equipped with two lasers (blue, 488; yellow/green, 561 nm; all tuned at 100 mW). Cell sorting was performed with 96-99% purity. Sorted CD3+CD8+ T cells were immediately loaded on ddSEQ single-cell isolator (Bio-Rad Laboratories) to isolate single cells and barcode single cells. sc-RNA-seq libraries were prepared by using the Illumina Bio-Rad SureCell WTA 3' Library Prep Kit (Illumina, San Diego, CA, manufactured for Bio-Rad) following manufacturer's instructions. Briefly, after barcoding, RNA was reverse transcribed and cleaned up. Then, second strand cDNA was synthesized and tagmented. Tagmented DNA was amplified and final indexed libraries were quantified by using the high sensitivity DNA kit (Agilent) on a bioanalyzer (Agilent). Sequenced libraries were loaded on an Illumina MySeq.

Data analysis

Flow Cytometry Standard (FCS) 3.0 files were analysed using FlowJo software V 9.6. Aggregates and dead cells were removed from the analyses and identify CD3+ CD8+ T cells were gated. 10,000 CD8+ T cells per sample were exported and biexponentially transformed in FlowJo V10. Further analyses were performed by a custom-made script that makes use of Bioconductor libraries and R statistical packages 4. Data were analyzed using the Phenograph algorithm coded in the Cytofkit package (version 1.6.5) in R (version 3.3.3).

Downstream analysis for sc-RNAseq was performed in R using Seurat v3.0. Principal components were selected using the jackstraw and Elbow methods. The dimensional reduction was performed using Uniform Manifold Approximation and Projection (UMAP) on the previously selected principal components. Unsupervised clustering was performed by finding the K-nearest neighbors (KNN) and then, to group the cells, a modularity optimization-based algorithm was applied. Differentially expressed genes were identified using the FindAllMarkers function.

For manuscripts utilizing custom algorithms or software that are central to the research but not yet described in published literature, software must be made available to editors and reviewers. We strongly encourage code deposition in a community repository (e.g. GitHub). See the Nature Research guidelines for submitting code & software for further information.

Data

Policy information about availability of data

All manuscripts must include a data availability statement. This statement should provide the following information, where applicable:

- Accession codes, unique identifiers, or web links for publicly available datasets
- A list of figures that have associated raw data
- A description of any restrictions on data availability

Data availability
The source data underlying Figs. 1–4 are provided as a Source Data file. Original.fcs files concerning cytofluorimetric analysis (Figs. 1 and 4) are deposited at the flowrepository.org in the following folders: FR-FCM-Z36A, https://flowrepository.org/id/FR-FCM-Z36A; MAIT: FR-FCM-Z36B, https://flowrepository.org/id/FR-FCM-Z36B.
The scRNA-seq data have been deposited in GEO under the accession code GSE166181 at this link https://www.google.com/url?q=https://www.ncbi.nlm.nih.gov/geo/query/acc.cgi?acc%3DGSE166181&source=gmail-imap&ust=1613062707000000&usg=AOvVaw3pkgx6jlI_SmKrWhetSRIW.
Code availability: https://github.com/DomenicoSkyWalker89/CD8-T-lymphocytes-MAIT

Field-specific reporting

Please select the one below that is the best fit for your research. If you are not sure, read the appropriate sections before making your selection.

[x] Life sciences [ ] Behavioural & social sciences [ ] Ecological, evolutionary & environmental sciences

For a reference copy of the document with all sections, see nature.com/documents/nr-reporting-summary-flat.pdf

Life sciences study design

All studies must disclose on these points even when the disclosure is negative.

Sample size

Due to the design of the study, i.e., the analysis of CD8+ T cells in patients with metastatic melanoma and their follow up for at least 6 months, and according to the permission of the local Ethical Committee, we decided to enroll all patients who arrived to our attention. However, according to our previous and large experience on the analysis of T cells, and to the prediction of response or not to therapy (that was estimated to be around 50/50) a sample size of 28 patients was considered sufficient to detect relevant differences.

Data exclusions

No data excluded.

Replication

Thawed PBMC isolated from the same healthy donors were used for flow cytometry experiments internal control, in order to avoid batch effect that could occur performing experiments in different days.

Randomization

No randomization.

Blinding

The investigators and the patients were not blinded.

Reporting for specific materials, systems and methods

We require information from authors about some types of materials, experimental systems and methods used in many studies. Here, indicate whether each material, system or method listed is relevant to your study. If you are not sure if a list item applies to your research, read the appropriate section before selecting a response.

Materials & experimental systems

n/a	Involved in the study
☐	☒ Antibodies
☒	☐ Eukaryotic cell lines
☒	☐ Palaeontology and archaeology
☒	☐ Animals and other organisms
☐	☒ Human research participants
☐	☒ Clinical data
☒	☐ Dual use research of concern

Methods

n/a	Involved in the study
☒	☐ ChIP-seq
☐	☒ Flow cytometry
☒	☐ MRI-based neuroimaging

Antibodies

Antibodies used

All antibodies used in this study are against human proteins.

Reagents for the 28-color panel used for the characterization of T cells (Specificity, Dye, Clone, Vendor, Catalog Number, Lot number, uL used in a volume of 100 uL): AQUA, BV510, NA, Biolegend, 423102, B244918, 0.06; CCR7, BB660, 150503, Becton Dickinson (BD), 625454, 7136810, 1.25; CXCR6, BV421, 13B, 1E5, BD, 566008, 7131852, 2.50; CD194, PE-CF594, 1G1, BD, 565391, 7276847, 0.30; CD25, BB790, M-A251, BD, 353736, 7150757, 2.5; CD244, PC5.5, C1.7, Beckman Coulter (BC), B21171, 16, 1.25; CD98, BB515, UM7F8, BD, 565103, 7283973, 1.25; CD28, BV786, CD28.2, Biolegend, 302950, B249581, 0.6; CD39, APC-H7, A1, Biolegend, 328226, B246491, 1.25; CD127, APC-R700, HIL-7R-M21, BD, 565185, 7221803, 2.5; CD38, BV711, HIT2, Biolegend, 303528, B238633,2.5; CD272, (BTLA), BV650, J168-540, BD, 564803, 7103949, 0.60; CD57, BV605, NK-1, BD, 563895, 7018755, 1.25; CD27, BV570, O323, Biolegend, 302825, B244350, 1.25; CD71, PE-CY5, M-A712, BD, 551143, 7058789, 0.60; CD8, BUV805, SK1, BD, 564912, 7311599, 1.25; CD95, BUV737, DX2, BD, 564710, 7032582, 0.63; HLA-DR, BUV661, G46-6, BD, 565073, 7249926, 0.03; CD4, BUV615, SK3, BD, 624297, NA, 0.31; CD45RA, BUV563, HI100, BD, 565702, 7219651, 1.25; CD3, BUV496, UCHT1, BD, 564809, 7312719, 2.50; ICOS, CD278, BUV395, DX29, BD, 564777, 7263764, 2.50; PD-1, PE, EH12.1, BD, 570695, NA, 2.5; IgG4, PE, HP6025, Southern Biotech, 9200-09, B3317-YD17B, 1.25; TBET, PE-CY7 4B10, eBioscience, 25-5825-80, 4286798, 0.15; GRANULYSIN, APC, DH2, Biolegend, 348006, B192033, 5.00; KI67, BV480, B56, BD, 566109, 7254647, 5.00.

mAbs used in flow cytometry panel used for the detection of MAIT cells (Specificity, Dye, Clone, Vendor, Catalog Number, Lot number, uL used in a volume of 100 uL): PromoFluor-840, Maleimide, NA, Promokine, PK-PF840-3-05, 429P017, 0.3; CXCR4, BV421, 12G5, BD, 562448, 4003566, 1.25; CCR7, BV605, G043H7, Biolegend, 353224, B284011, 2.5; CD3, AF700, UCHT1, Biolegend, 300424, B279942, 0.6; CD8a, APC-CY7, RPA-T8, Biolegend, 301016, B274260, 1.5; CD161, PC7, B30631, Beckman Coulter, B191B8, 200023, 2.5; TCR72, PE, 3C10, Biolegend, 351706, B292236, 1.25; CD69, FITC, FN50, Biolegend, 310904, B290844, 0.6; CD95, BUV395, DX2, BD, 740306, 9269965,0.6; CD38, BUV496, HIT2, BD, 564658, 8339559, 1.25; HLA-DR, BUV661, G46-6, BD, 565073, 7249926, 0.3; CD127, BV650, A019D5, Biolegend, 351325, B238890, 0.6; CD25, BV785, BC96, Biolegend, 302638, B237289, 1.25; CD45RO, PC5.5, UCHL1, Beckman Coulter, B30638, 200022, 10; Ki67, BV480, B56, BD, 566109, 7254647, 5; GRANULYSIN, AF647, DH2, Biolegend, 348006, B192033, 5.

mAbs used for investigating the function of MAIT cells (Specificity, Dye, Clone, Vendor, Catalog Number, Lot number, uL used in a volume of 100 uL): Live Dead, AQUA, N/A, ThermoFisher, L34965, 2069643, 1.25; CD4, AF700, RPA-T4, Biolegend, 300526, B274110, 0.6; CD8, APC-Cy7, RPA T8, Biolegend, 301016, B274260, 0.6; CD161, PE-Cy7, HP-3G10, Biolegend, 339918, B204086, 0.6; TCR 7.2, PE, 3C10, Biolegend, 351706, B292236, 1.25; CD3, PE-Cy5, UCHT1, Biolegend, 300410, B252119, 0.6; TNF, BV605, MAb11, Biolegend, 502936, B282176, 3.75; IFN-g, FITC, B27, Biolegend, 506504, B286029, 2.5; GRZB, BV421, QA18A28, Biolegend, 396414, B296875, 2.5; GRZA, APC, CB9, Biolegend, 507220, B277773, 5.

Validation

All antibodies used in this study are commercially available; all have been validated against human antigens by the manufacturers and used by an uncountable number of other scientists and laboratory personnel. Likewise, we titrated these antibodies according to our own staining conditions.

April 2020

Human research participants

Policy information about studies involving human research participants

Population characteristics	The study was conducted on 28 patients with metastatic melanoma treated with standard-of-care nivolumab or pembrolizumab. According to the RECIST, responders (n=17) were defined as patients with complete response (CR), partial response (PR), stable disease (SD), or mixed response (MR) of greater than 6 months with no progression, and non-responders (n=11) as patients with progressive disease (PD). In particular, among responders, 41.2% had CR, 35.3% had a PR, 17.6% had SD, and 5.9% (which corresponds to one patient) had a MR. The mean age of the total cohort was 71 years. No patient had previously received other therapies.
Recruitment	Patients admitted to the University Hospital in the period January 2017-January 2020 were recruited (time 0). They all received a diagnosis of metastatic melanoma, and have been followed by the Oncology department.
Ethics oversight	All human blood samples (up to 30 mL) were obtained via informed consent through the Azienda Ospedaliero Universitaria di Modena and Reggio Emilia. Approval of study protocols was obtained by the ethical committee (Prot AOU 0005400/18).

Note that full information on the approval of the study protocol must also be provided in the manuscript.

Clinical data

Policy information about clinical studies

All manuscripts should comply with the ICMJEguidelines for publication of clinical research and a completedCONSORT checklist must be included with all submissions.

Clinical trial registration	n/a
Study protocol	n/a
Data collection	n/a
Outcomes	n/a

Flow Cytometry

Plots

Confirm that:

- [x] The axis labels state the marker and fluorochrome used (e.g. CD4-FITC).
- [x] The axis scales are clearly visible. Include numbers along axes only for bottom left plot of group (a 'group' is an analysis of identical markers).
- [x] All plots are contour plots with outliers or pseudocolor plots.
- [x] A numerical value for number of cells or percentage (with statistics) is provided.

Methodology

Sample preparation	Peripheral blood mononuclear cells (PBMC) were isolated according to standard procedures and stored in liquid nitrogen until use. The whole experimental procedure is represented in Supplementary Figure 1.
Instrument	Samples were acquired on different flow cytometers, i.e.: FACS Symphony A5 (BD Biosciences) equipped with five lasers (UV, 350 nm; violet, 405 nm; blue, 488; yellow/green, 561 nm; red, 640 nm); Attune NxT (ThermoFisher) with 405, 488, 561, and 637 nm lasers; S3e cell sorter (Bio-Rad) with 488 and 561 nm lasers; Cytoflex LX (Beckman Coulter) with 355, 405, 488, 561, 638 and 808 nm lasers.
Software	Flow cytometry data were compensated in FlowJo by using single stained controls (BD Compbeads incubated with fluorochrome-conjugated antibodies) and Fluorescence minus one (FMO) approach.
Cell population abundance	Sorting of CD8+ T cells were performed and purity was checked after sorting. Purity was always >96%.
Gating strategy	Gating strategies have been performed according to the state of art (Cossarizza A. et al., Guidelines for the use of flow cytometry and cell sorting in immunological studies - Second Edition. European Journal of Immunology 49(10): 1457-1973, 2019) and are shown in Supplementary Figure 1, 5, 6.

- [x] Tick this box to confirm that a figure exemplifying the gating strategy is provided in the Supplementary Information.
